# Supplementary material for: Ubiquitome profiling reveals a regulatory pattern of UPL3 with UBP12 on metabolic-leaf senescence
Source: Life Sci Alliance. 2022 Aug 4;5(12):e202201492. doi: 10.26508/lsa.202201492 (PMC9354775; doi:10.26508/lsa.202201492)

Fig2C

WT, upl3-1, upl3-3, oeUPL3-24, oeUPL3-39, upl5

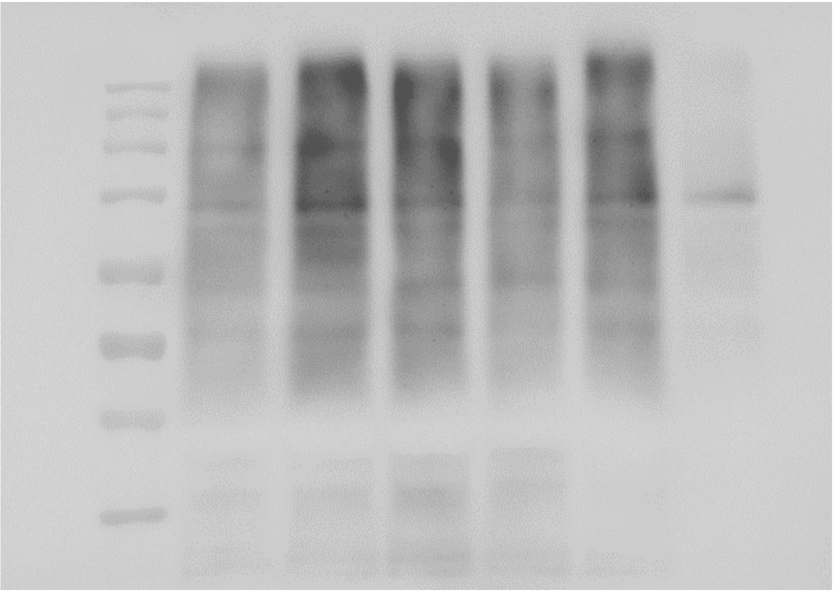

α-Ubn

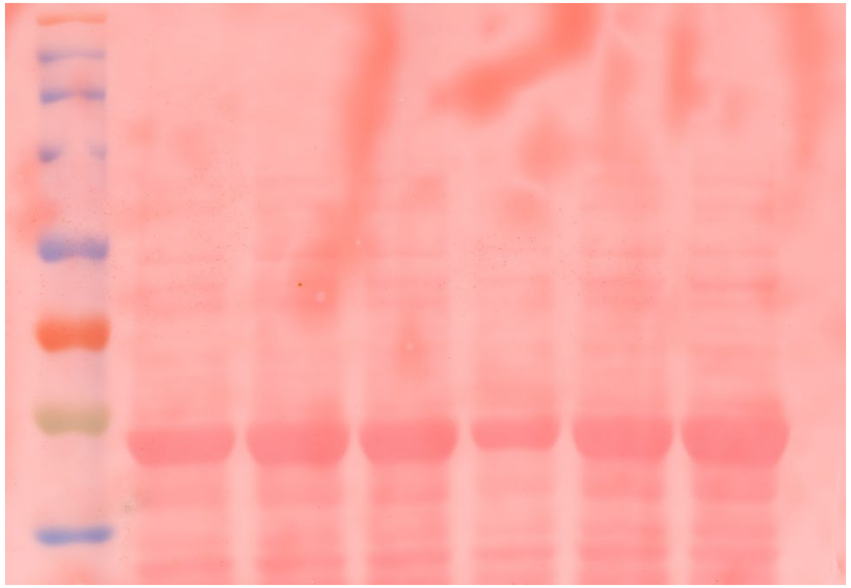

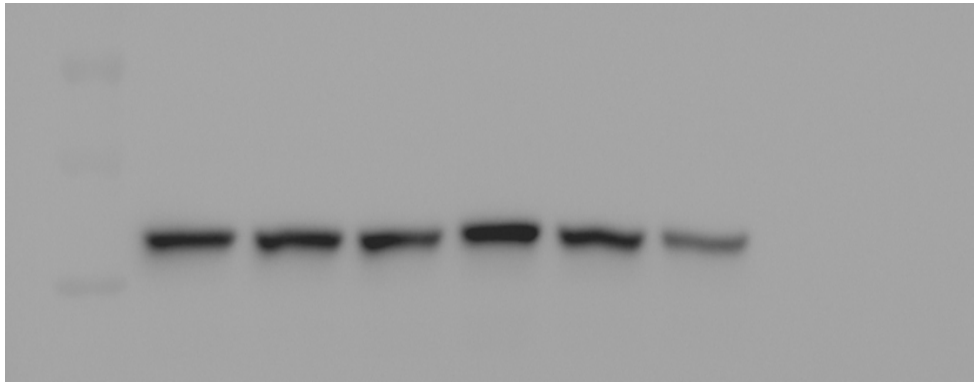

α-tubulin

Fig5A

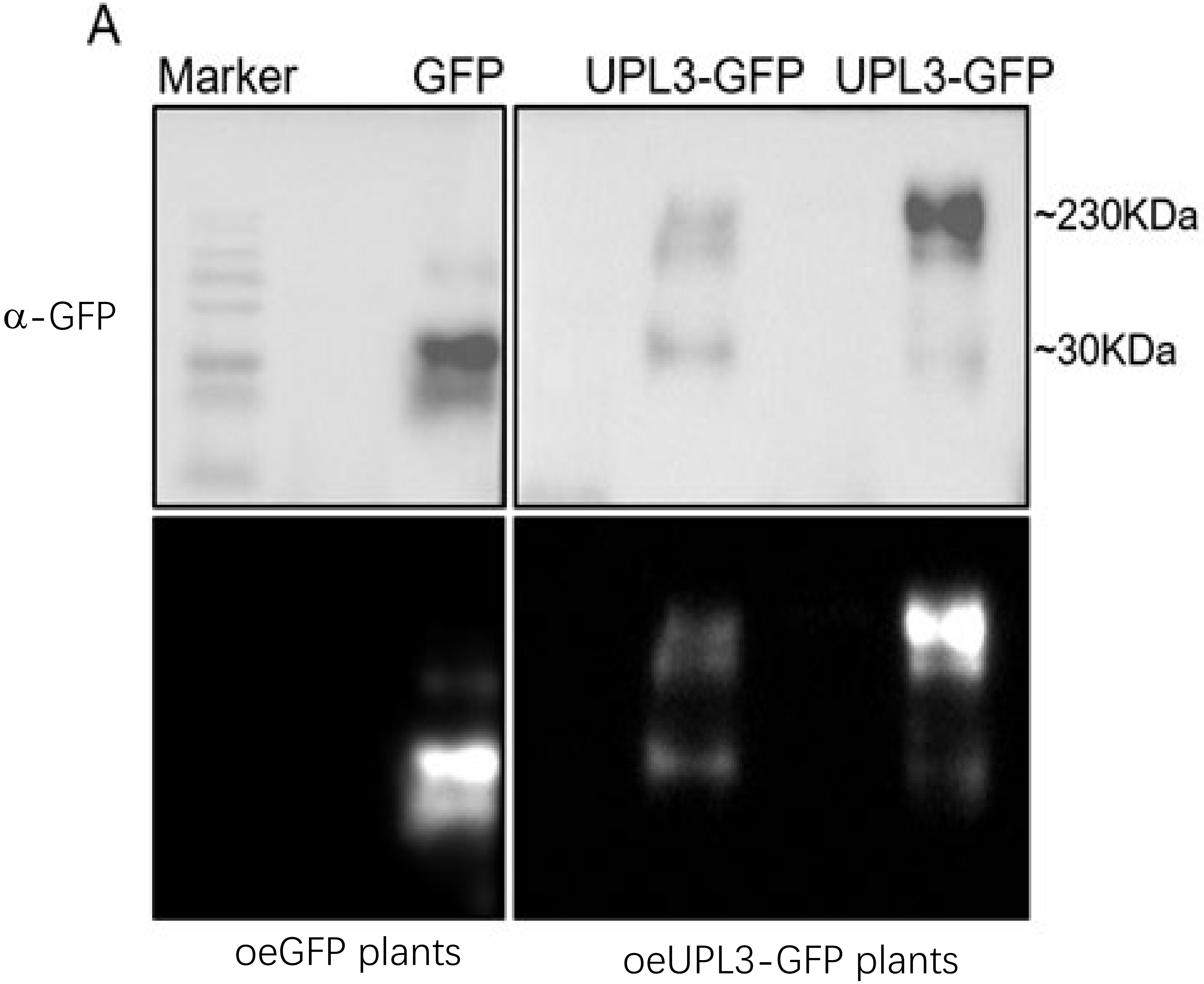
A
Marker
GFP
UPL3-GFP
UPL3-GFP
α-GFP
~230KDa
~30KDa
oeGFP plants
oeUPL3-GFP plants

Fig6F-input-GFP, PP2C, HXK1,

oeUPL3-GFP, oeUPL3-GFP, WT, oeUBP12, oeUBP12, WT

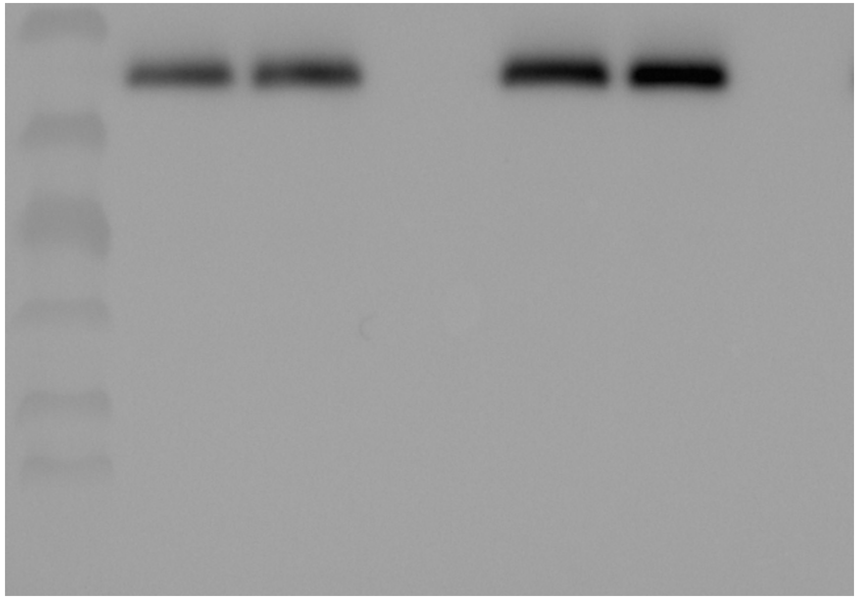

α-GFP

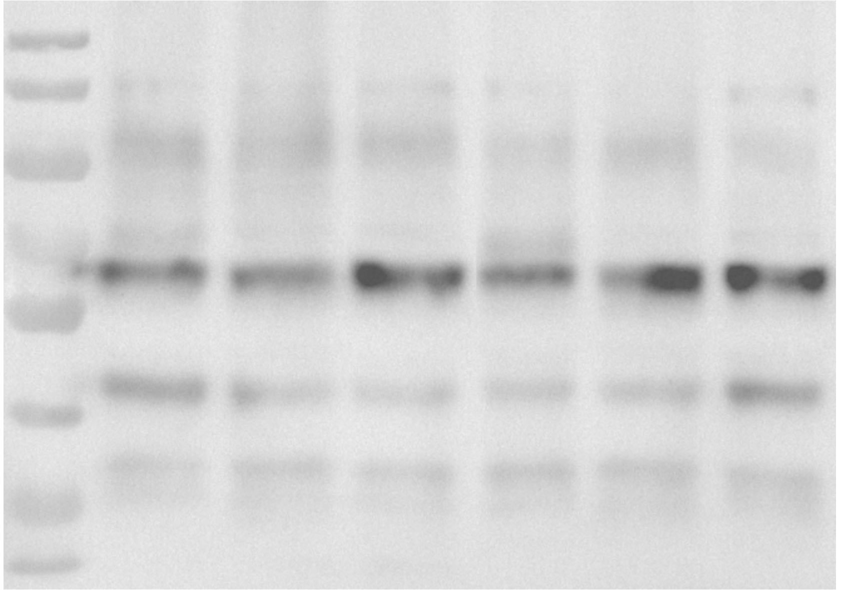

α-HXK1

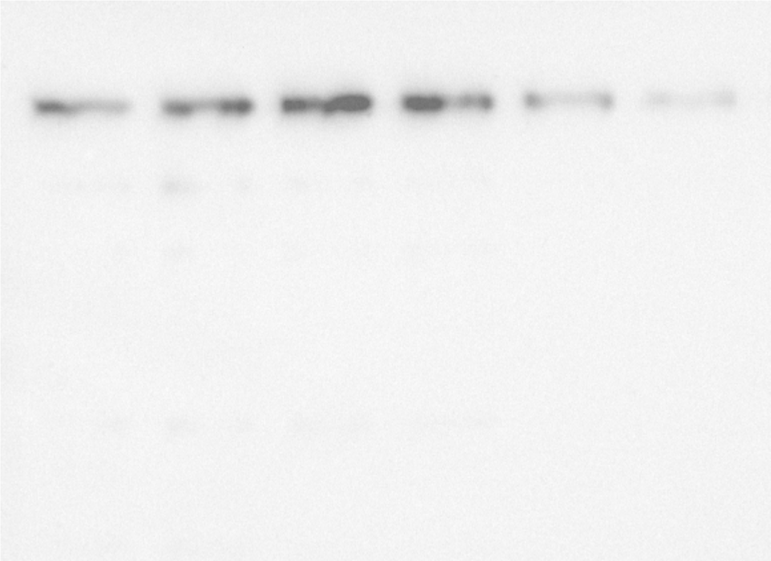

α-PP2C

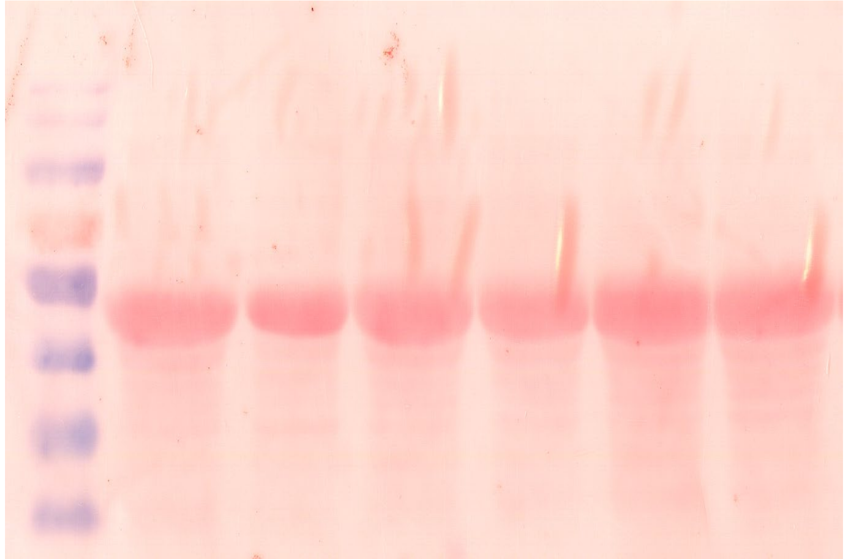

Fig6F-IP-GFP, PP2C, HXK1,

oeUPL3-GFP, oeUPL3-GFP, WT, oeUBP12-GFP, oeUBP12-GFP, WT

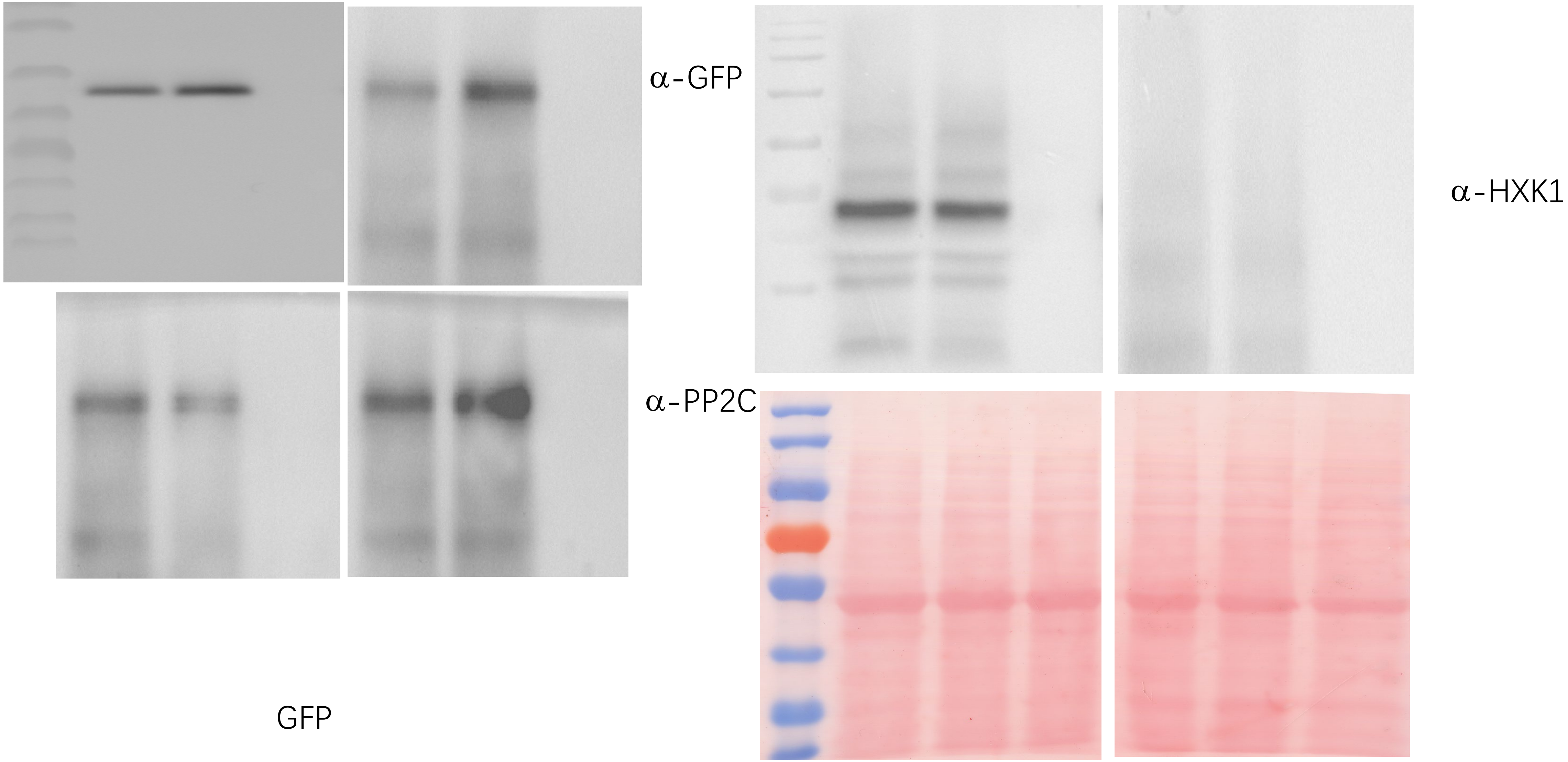

Fig7D

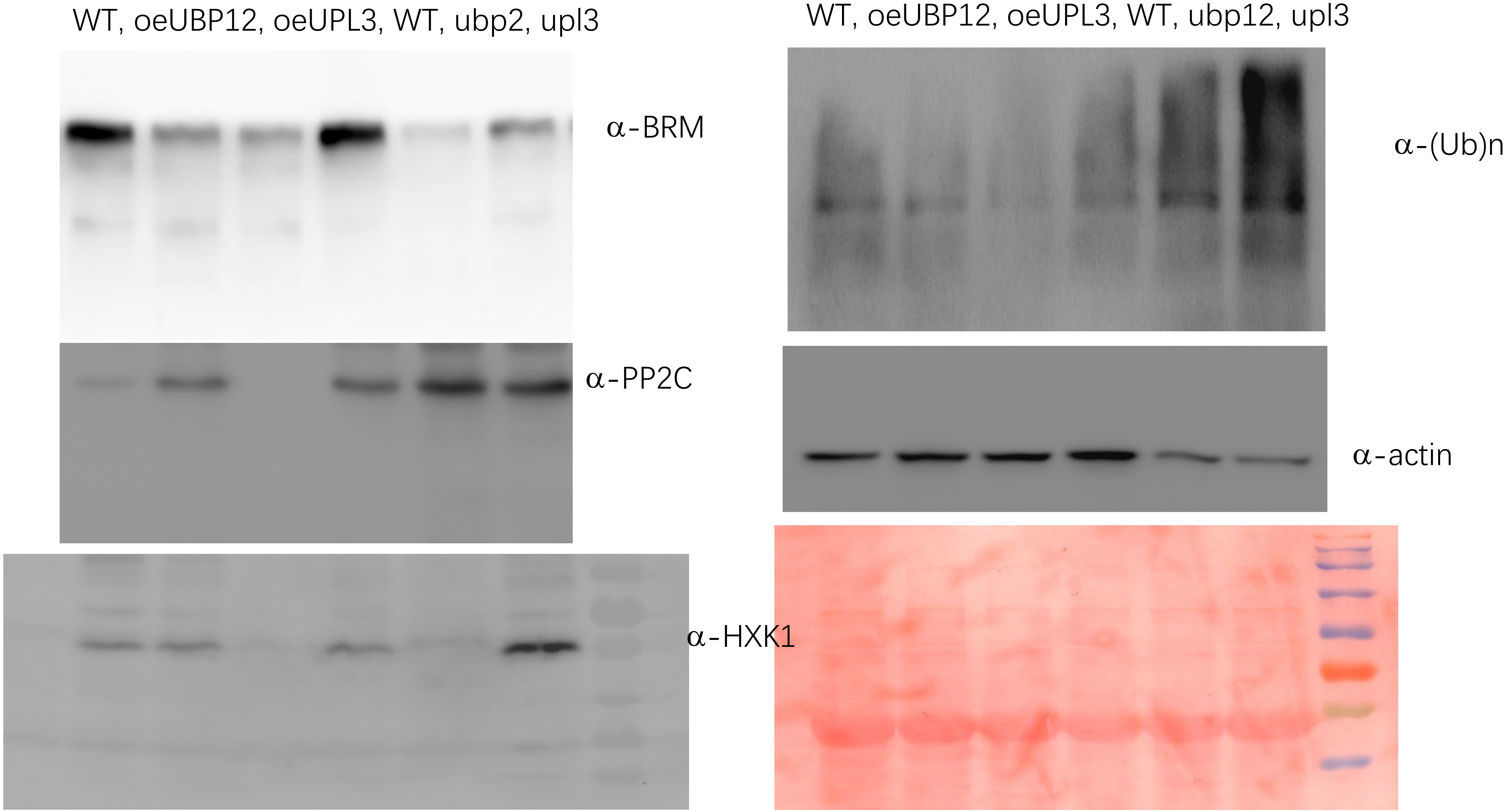

WT, oeUBP12, oeUPL3, WT, ubp2, upl3
α-BRM
α-PP2C
α-HXK1
WT, oeUBP12, oeUPL3, WT, ubp12, upl3
α-(Ub)n
α-actin

Fig 7E-PartI

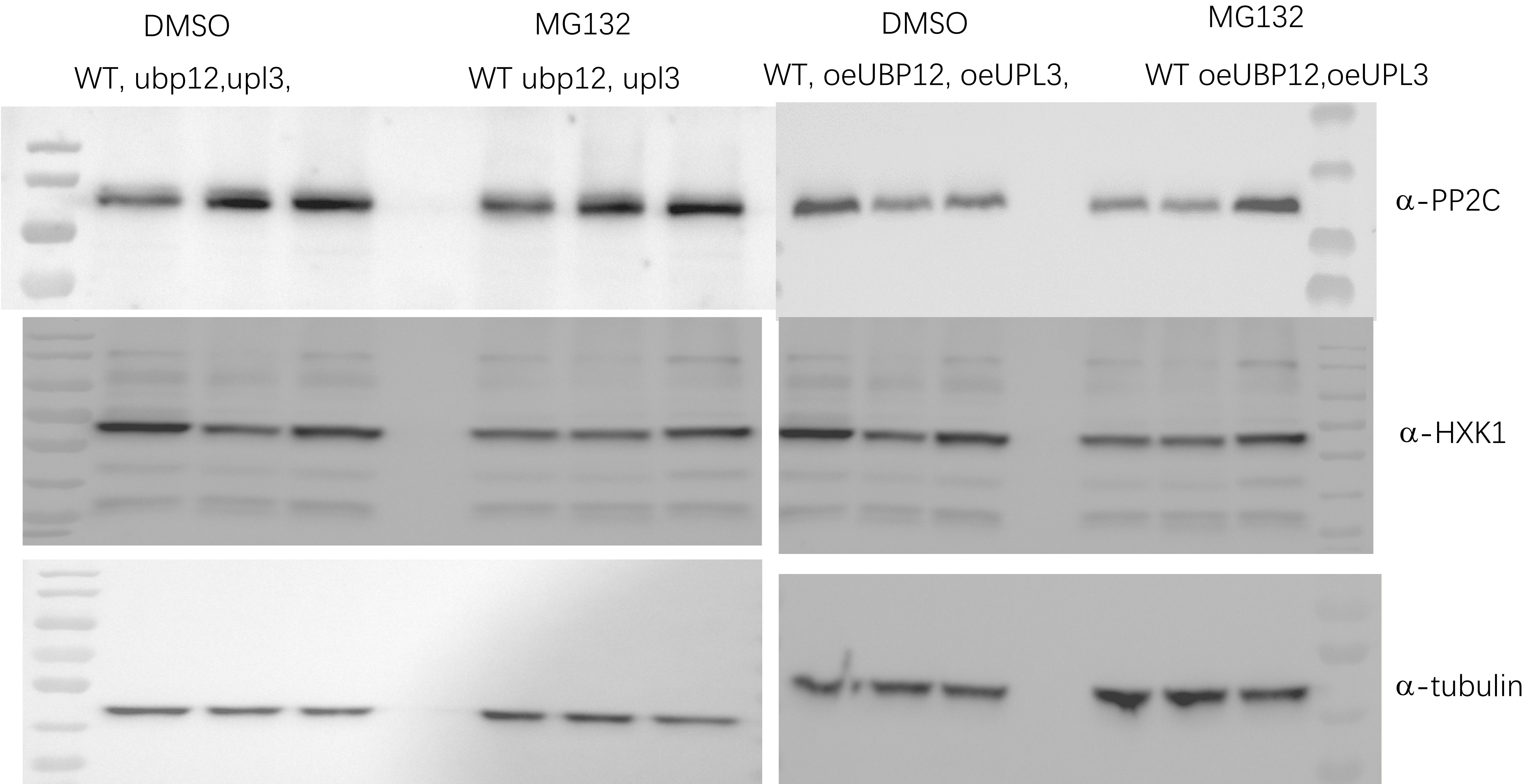
DMSO
WT, ubp12,upl3,
MG132
WT ubp12, upl3
DMSO
WT, oeUBP12, oeUPL3,
MG132
WT oeUBP12,oeUPL3
α-PP2C
α-HXK1
α-tubulin

Fig 7E-partII

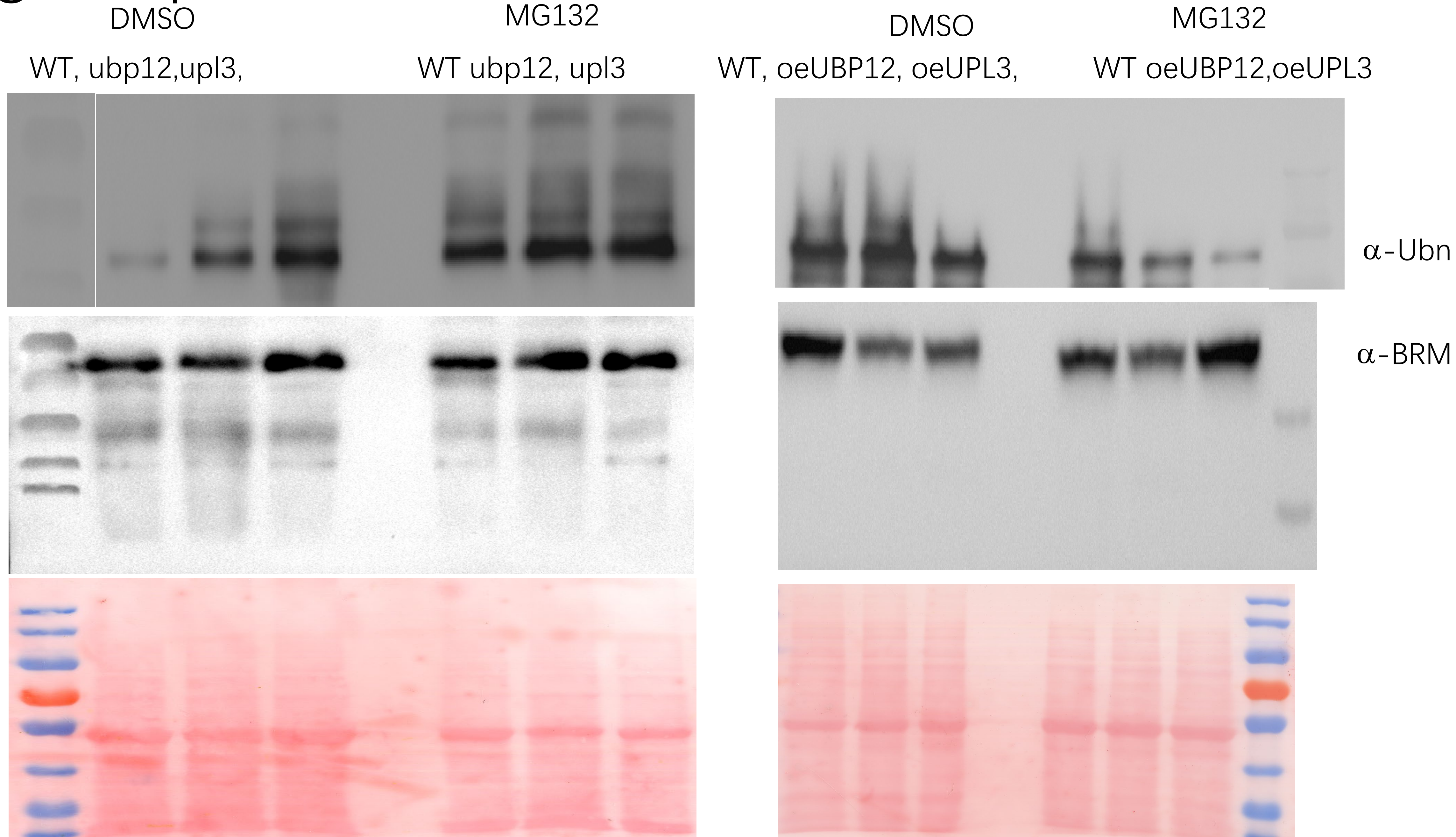

Supplement: Supplementary file 3 [file LSA-2022-01492_SdataF2_F5_F6_F7.pdf]
