# Supplementary material for: Ubiquitome profiling reveals a regulatory pattern of UPL3 with UBP12 on metabolic-leaf senescence
Source: Life Sci Alliance. 2022 Aug 4;5(12):e202201492. doi: 10.26508/lsa.202201492 (PMC9354775; doi:10.26508/lsa.202201492)
Supplement: Supplementary file 8 [file LSA-2022-01492_TableS1.docx]

Supplementary Table S1 Primers used in the construction of binary vectors

| Vector | Primer | Sequence（5’-3’） | Method |
| --- | --- | --- | --- |
| ProACTIN2:UPL3-GFP | P1-FP | TGACCTCGAGACTAGTATGGAAACTCGGAGCCGCAAG | Infusion |
|  | P1-RP | GTGGTTGAAGCTGGAGTTGAC |  |
|  | P2-FP | TCCAGCTTCAACCACTCGT |  |
|  | P2-RP | GTGGAGGTCCCCCGGGTGAGAGGTCGAACGATCCTTGC |  |
| ProACTIN2:UPL3(m)-GFP | P3-FP | AGTGTCATGACTGGCGCAAACTAC | Infusion |
|  | P3-RP | GTAGTTTGCGCCAGTCATGACACT |  |
| ProUPL3:UPL3-GFP | P5-FP | ATGGAAACTCGGAGCCGCAAG | Infusion |
|  | P2-RP | GTGGAGGTCCCCCGGGTGAGAGGTCGAACGATCCTTGC |  |
|  | P4-FP | CAATGATTACGAATTCCTGCAGTCCGCTGGCAGATAGTTTCA |  |
|  | P4-RP | GTCCTTGTAGTCCATTCTAGACGGCTCCGAGTTTCCATACA |  |
| ProUPL3:UPL3(m)-GFP | P5-FP | ATGGAAACTCGGAGCCGCAAG | Infusion |
|  | P2-RP | GTGGAGGTCCCCCGGGTGAGAGGTCGAACGATCCTTGC |  |
|  | P4-FP | CAATGATTACGAATTCCTGCAGTCCGCTGGCAGATAGTTTCA |  |
|  | P4-RP | GTCCTTGTAGTCCATTCTAGACGGCTCCGAGTTTCCATACA |  |
