# Supplementary material for: Ubiquitome profiling reveals a regulatory pattern of UPL3 with UBP12 on metabolic-leaf senescence
Source: Life Sci Alliance. 2022 Aug 4;5(12):e202201492. doi: 10.26508/lsa.202201492 (PMC9354775; doi:10.26508/lsa.202201492)
Supplement: Supplementary file 9 [file LSA-2022-01492_TableS2.docx]

Supplementary Table S2 Primers used in mutant plants identification.

| Mutant plants | Primer | sequence（5’-3’） |
| --- | --- | --- |
| upl3-1(SALK_015334) | FP | GATATGTCAGCTGTTGAGGGC |
|  | RP | TAGGGACTTGCATGGACGTAC |
| upl3-3(SALK_117247) | FP | TGATAAGCCGAACTCACTTGG |
|  | RP | GGTAGCACCAAGAGGGAAGAC |
| P1 | FP | GGTCGAGGCTTTAACCCAGC |
| P2 | RP | GGTCGAGGCTTTAACCCAGC |
| P3 | FP | TCCAGCTTCAACCACTCGT |
| P4 | RP | GGTAGCACCAAGAGGGAAGAC |
| P5 | FP | GGAATTCACGCTACCTGGCT |
| P6 | RP | CCCTTCGTTGATGGCGTAGA |
| GAPC2 | FP | ACCACTGTCCACTCTATCACTGA |
|  | RP | TGAGGGATGGCAACACTTTCCC |
