# Supplementary material for: Ubiquitome profiling reveals a regulatory pattern of UPL3 with UBP12 on metabolic-leaf senescence
Source: Life Sci Alliance. 2022 Aug 4;5(12):e202201492. doi: 10.26508/lsa.202201492 (PMC9354775; doi:10.26508/lsa.202201492)
Supplement: Supplementary file 10 [file LSA-2022-01492_TableS3.docx]

Supplementary Table S3 Primers used in RT-qPCR

| genes | Access number | Primer | sequence（5’-3’） |
| --- | --- | --- | --- |
| C2H2ZF | AT1G30970 | FP | TGCACGATGAAACCAGCCAGATG |
|  |  | RP | TCTTTGACGCTGGTTTGGTTTGG |
| ZF15 | AT2G01940 | FP | TTGTTCCGCACGGAGAGTTCAC |
|  |  | RP | ACTGTTACCGCGGTTTGTGGTG |
| WRKY38 | AT5G22570 | FP | ACTGCGAAGCAAGAAAGCATGAAC |
|  |  | RP | TGGTGGCCAAAGTAAGTGGTTCG |
| WRKY63 | AT1G66600 | FP | AACATCGATCACAAGGCTGTGG |
|  |  | RP | TCTTGAGGATGTTAGCGCATCCC |
| WRKY30 | AT5G24110 | FP | TCTCGGAGCCAAATTTCCAAGAGG |
|  |  | RP | TCCTCGGTAACTGATCTCAAGGAG |
| WRKY75 | AT5G13080 | FP | AGTGGACCAAGAAGTGGTCGTG |
|  |  | RP | TTCTCGATGGGATGCGAATGCAC |
| WRKY53 | AT4G23810 | FP | CAGACGGGGATGCTACGG |
|  |  | RP | GGCGAGGCTAATGGTGGTG |
| WRKY51 | AT5G64810 | FP | GACGGGTCATCGAGTTGCATTTAG |
|  |  | RP | ACCGAGCAACCTTCACTTGAGC |
| NAC042 | AT2G43000 | FP | TCTCCAGCTCAACAAGCAGAGG |
|  |  |  | CGGTTTGGTGGTAAGATGGTTGGG |
| bZIP1 | AT5G49450 | FP | TTCTCCCACTTTCCTTATTTTCG |
|  |  | RP | GTGATTTGCGAGGATCAAGAG |
| MYB46 | AT5G12870 | FP | CAACAGGTGGTCTCAGATTGCAG |
|  |  | RP | GCTGTGTTGGGTGATGAGGATGAG |
| ANAC041 | AT2G33480 | FP | CCGATCCATGGGATTTACCAGGTG |
|  |  | RP | TCGCTTTCCAATAACCCGAACCG |
| ANAC004 | AT1G02230 | FP | GGATCTGAGGGTTCGTCGTTTCAC |
|  |  | RP | TCCCTGCACATTAGCAAAGACACC |
| ANAC047 | AT3G04070 | FP | AACTCTTCTAGGAGCGGTGGTAGC |
|  |  | RP | GGCAAAGAACCCAATCATCCAGTC |
|  |  |  |  |
