# Supplementary material for: Ubiquitome profiling reveals a regulatory pattern of UPL3 with UBP12 on metabolic-leaf senescence
Source: Life Sci Alliance. 2022 Aug 4;5(12):e202201492. doi: 10.26508/lsa.202201492 (PMC9354775; doi:10.26508/lsa.202201492)
Supplement: Supplementary file 11 [file LSA-2022-01492_TableS4.docx]

Supplementary Table S4 Primers used in yeast-two hybrid assays

| Vector | Primer | sequence（5’-3’） | Method |
| --- | --- | --- | --- |
| pGADT7 AD-BRM | FP | GGCCAGTGAATTCCATATGCAATCTGGAGGCAGTGG | Infusion |
|  | RP | ATTCATCTGCAGCTCGAGCTCGTAAATGGCTAGGCCGTCTTTTAC |  |
| pGADT7 AD -BRM-N | FP | GGCCAGTGAATTCCATATGCAATCTGGAGGCAGTGG | Infusion |
|  | RP | ATTCATCTGCAGCTCGAGCTCGTCTGATCACAACTTCTTCTCGAGC |  |
| pGADT7 AD -BRM-M | FP | GGCCAGTGAATTCCATAATAGATTTACGGAAATGAATGCG | Infusion |
|  | RP | ATTCATCTGCAGCTCGAGCTCGAGTTGTCCTTTGGTCAAAACGAC |  |
| pGADT7 AD -BRM-C | FP | GGCCAGTGAATTCCATCATGAAGAGCGACGAATGACAT | Infusion |
|  | RP | ATTCATCTGCAGCTCGAGCTCGTAAATGGCTAGGCCGTCTTTTAC |  |
| pGADT7 AD -HDA15 | FP | GGCCGAATTCATGGTTGTAGAAACTATCGAGAGGTC | Ligation |
|  | RP | GCCGCTGCAGGAGCTCCGACGGATTAGGAAGAATGCTTTC |  |
| pGBKT7 BD-UPL3-I | FP | ATGGCCATGGAGGCCATGGAAACTCGGAGCCGC | Infusion |
|  | RP | CCGCTGCAGGTCGACGCCCAGAACCCAACAGAATATCC |  |
| pGBKT7 BD -UPL3-III | FP | ATGGCCATGGAGGCCATGGAAACTCGGAGCCGC | Infusion |
|  | RP | CCGCTGCAGGTCGACGAAGGACAACAGGAAAGCGCTC |  |
| pGBKT7 BD -UPL3 | FP | ATGGCCATGGAGGCCATGGAAACTCGGAGCCGC | Infusion |
|  | RP | CCGCTGCAGGTCGACGGATCCCTGAGAGGTCGAACGATCCTTG |  |
| pGBKT7 BD -UPL5 | FP | TCCACCCGGGAATGACTCTAAGCCGTTCATCAGC | Ligation |
|  | RP | CCCACCCGGGCCATTTACCGAAACTGGAGCTGAC |  |
| pGBKT7 BD -HDA15 | FP | GGCCGAATTCATGGTTGTAGAAACTATCGAGAGGTC | Ligation |
|  | RP | GCCGCTGCAGGAGCTCCGACGGATTAGGAAGAATGCTTTC |  |
