# Supplementary material for: Ubiquitome profiling reveals a regulatory pattern of UPL3 with UBP12 on metabolic-leaf senescence
Source: Life Sci Alliance. 2022 Aug 4;5(12):e202201492. doi: 10.26508/lsa.202201492 (PMC9354775; doi:10.26508/lsa.202201492)
Supplement: Supplementary file 12 [file LSA-2022-01492_TableS5.docx]

Supplementary Table S5 The description of Dataset in the ProteomeXchange Consortium via the PRIDE partner repository with the dataset identifier PXD027037.

| File names | plants | sample* |
| --- | --- | --- |
| 71147LPUb_A1 | Col-0 | Rep1-A1 |
| 71147LPUb_A2 | Col-0 | Rep2-A2 |
| 71147LPUb_B1 | upl3-1 | Rep1-B1 |
| 71147LPUb_B3 | upl3-1 | Rep2-B3 |

*The rosette leaves of ten 6-week-old upl3-1 and wildtype plants were harvested, pooled and ground in liquid nitrogen and then transferred to three tubes of 5 mL centrifuge tube as one biological replicate. Total three biological replicates were setup. Due to one replicate could not get high quality of data, two replicates of wildtype plant (rep1-A, rep2-A) and upl3 plants (rep1-B, rep2-B) of datasets were used to analysis.
